# Hypoglycemic Syndrome in a Patient with Proinsulin-Only Secreting Pancreatic Adenoma (Proinsulinoma)

**DOI:** 10.1155/2011/930904

**Published:** 2011-07-03

**Authors:** Gian Paolo Fadini, Alberto Maran, Anna Valerio, Francesco Meduri, Mariarosa Pelizzo, Diego Miotto, Cristiano Lanza, Giuseppe Altavilla, Angelo Avogaro

**Affiliations:** ^1^Department of Clinical and Experimental Medicine, Metabolic Division, Medical School, University of Padova, 35100 Padova, Italy; ^2^Department of Medical and Surgical Sciences, Medical School, University of Padova, 35100 Padova, Italy; ^3^Department of Medical Diagnostic Sciences and Special Therapies, Medical School, University of Padova, 35100 Padova, Italy

## Abstract

We describe an unusual case of hypoglycemic syndrome in a 69-year old woman with a proinsulin-only secreting pancreatic endocrine adenoma. The clinical history was highly suggestive of an organic hypoglycemia, with normal or relatively low insulin concentrations and elevated proinsulin levels. Magnetic resonance and computed tomography of the abdomen showed a 1 cm pancreatic nodule and multiple accessory spleens. The diagnosis was confirmed by selective angiography, showing location and vascularization of the nodule, despite no response to intra-arterial calcium. After resection, the hypoglycemic syndrome resolved. The surgical specimen was comprised of a neuroendocrine adenomatous tissue with high proinsulin immunoreactivity. Study of this unusual case of proinsulinoma underlines (i) the need to assay proinsulin in patients with hypoglycemia and normal immunoreactive insulin, (ii) the differential diagnosis in the presence of accessory spleens, (iii) the unresponsiveness to intra-arterial calcium stimulation, and (iv) the extensive evaluation needed to reach a final diagnosis.

## 1. Introduction

The most common cause of organic fasting hypoglycemia in the adult is the presence of an insulin-producing pancreatic adenoma. The clinical diagnosis is relatively simple, but tumor localization is often challenging. In some cases, insulin concentrations are low despite a clinical history suggestive of pancreatic adenoma. In these cases, a proinsulinoma should be suspected. However, the rarity of this condition requires an extensive workup before reaching a final diagnosis.

## 2. Case Presentation

### 2.1. History

On August 2009, a 69-year old woman was admitted to the Division of Metabolic Diseases for recurrent episodes of hypoglycemia. About one month earlier, she began reporting symptoms of hypoglycemia (blurred vision, weakness, dizziness, sweating, mild tremor, hunger, paresthesia, and confusion), occurring in fasting conditions early in the morning and >4 hours after meals. On several occasions, during these episodes capillary glucose was found to be between 2.2 and 2.8 mmol/L (about 40–50 mg/dL), but she never lost consciousness. The patient had to snack several times a day to counter symptoms, and her body weight had increased by about 3 kg in 4 weeks. Her medical history included hypertension treated with an ACE inhibitor, remote Basedow's disease, and hypercholesterolemia on atorvastatin. She denied the use of insulin or oral antidiabetic medications. On admission, her vital signs were normal (blood pressure 140/80 mm Hg, heart rate 72 bpm), and physical examination was unremarkable.

### 2.2. Differential Diagnosis

**Figure 1:**
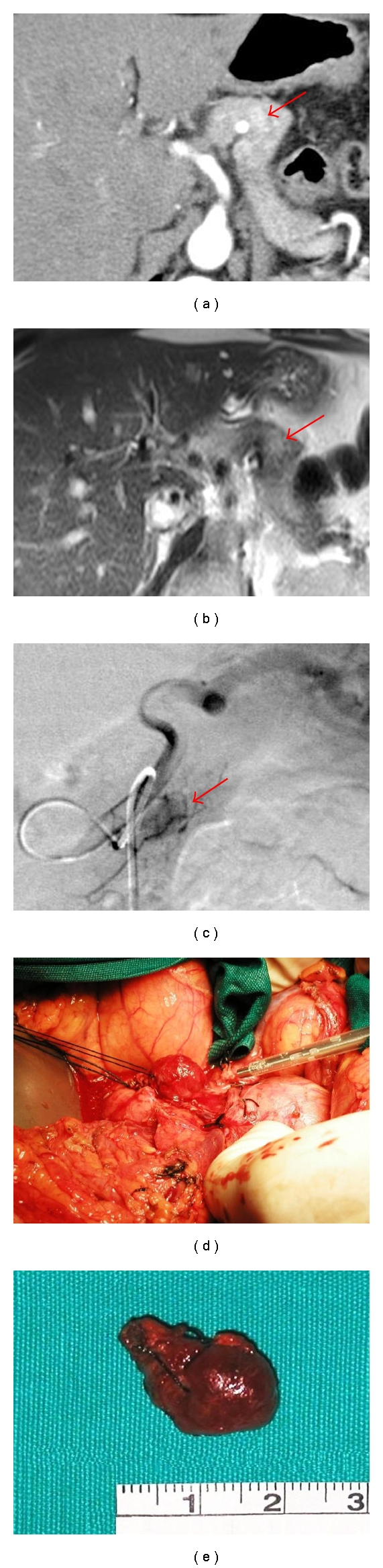
Imaging studies and intraoperative appearance. (a) A contrast-enhanced computed tomography slice of the upper abdomen, showing a mildly hyperintense nodule, about 1 cm in diameter, located between the body and the tail of the pancreas (red arrow). (b) A gadolinium-enhanced magnetic resonance slice of the upper abdomen, showing a mildly hyperintense nodule, about 1 cm in diameter between the body and the tail of the pancreas (red arrow), in the same location as identified by CT. (c) A digital subtraction angiography with the catheter positioned in the proximal splenic artery, showing nodular vascularization from the *arteria pancreatica magna* (red arrow). (d) Isolation and enucleation of the adenoma using the CUGA Excel system. (e) Macroscopic appearance of the adenoma soon after resection (scale bar in cm).

During the hospital stay, the patient was subjected to serial measurements of glucose, insulin and c-peptide serum concentrations before and after meals. Glucose levels were low before meals and within the normal range after meals; insulin and c-peptide concentrations physiologically increased after meals (Table 1). Fasting proinsulin concentration (measured by the EIA kit, DRG Instruments GmBH, Germany) was markedly elevated (7.2 pmol/L (64 pg/mL); normal 1.1 pmol/L (10 pg/L)). A 75 g oral glucose tolerance test revealed normal glucose tolerance (2 hour plasma glucose 7.4 mmol/L (134 mg/dL)) with no hypoglycemia up to 300 minutes, making reactive hypoglycemia less likely. Then, a 72-hour prolonged supervised fast was initiated: the test was interrupted after 23 hours with a typical Whipple triad (plasma glucose 1.9 mmol/L (34 mg/dL) and hypoglycemic symptoms resolved after ingestion of sugar and recovery of normal glucose levels); concomitantly, plasma insulin and c-peptide concentrations were 9 pmol/L (1.2 mU/L) and 0.26 nmol/L (0.8 *μ*g/L), respectively, thus ruling out typical insulin-producing adenoma. These findings were suggestive of an organic hypoglycemic syndrome. Although insulin and c-peptide were not always completely suppressed during hypoglycemic episodes, the low plasma insulin concentration and the elevated proinsulin levels indicated a proinsulinoma or a predominantly proinsulin-secreting adenoma. Then, the patient underwent a magnetic resonance scan of the abdomen, which revealed a rounded, solid nodule of about 10 mm in diameter located between the body and the tail of the pancreas. Given that MIR sometimes failed to exactly locate the pancreatic adenomas, this finding was confirmed by a CT scan, which also revealed the presence of multiple accessory spleens located within the spleen ilum and near the pancreatic tail (Figures 1(a) and 1(b)). An endoscopic ultrasound examination, with the US probe positioned in the large gastric curve, showed multiple accessory spleens near the tail of the pancreas that were mobile with changing decubitus, but was unable to locate the pancreatic nodule. During differential workup, the patient was advised to have multiple small meals and avoid sugars. Diazoxide was not administered because the patient could conduct a near-normal life by following dietary advices.

In light of instrumental findings, to rule out misdiagnosis of an accessory spleen as a pancreatic tail nodule, a selective angiography was performed with venous sampling [1]. Through femoral arteriotomy, a digital subtraction angiography was first performed with the catheter placed at the origin of the splenic artery. Then, a venous catheter was placed in the left suprahepatic vein, and stimuli with 0.05 mmol/Kg calcium gluconate were performed through the arterial catheter placed in the superior mesenteric artery, gastroduodenal artery proximal, and distal splenic arteries. Blood samples were drawn before and 30, 60, 120 seconds after the infusion of calcium gluconate. Digital angiography showed the presence of a 10-mm vascularized nodule in the pancreatic tail, served by the *arteria pancreatica magna*. Radioimmunological insulin and c-peptide determinations showed physiological values with no response to any of the calcium gluconate stimuli. Proinsulin concentrations were markedly elevated with no response to local stimuli (Table 2).

### 2.3. Treatment

Based on clinical, biochemical, and imaging studies, the patient was referred to a surgical division (F.M. and M.P.) with long-standing experience in the diagnosis and treatment of endocrine pancreatic tumors [2]. Under general anesthesia, the patient underwent laparotomy and surgical exploration of the pancreas; a 10 mm nodule was found where indicated by digital angiography and was enucleated from the pancreatic tail without local complications or bleeding. Enucleation was performed with care to spare the surrounding pancreatic tissue, using the CUSA EXcel (Tyco) ultrasonic surgical aspiration system (Figures 1(d) and 1(e)).

### 2.4. Tissue Analysis

**Figure 2:**
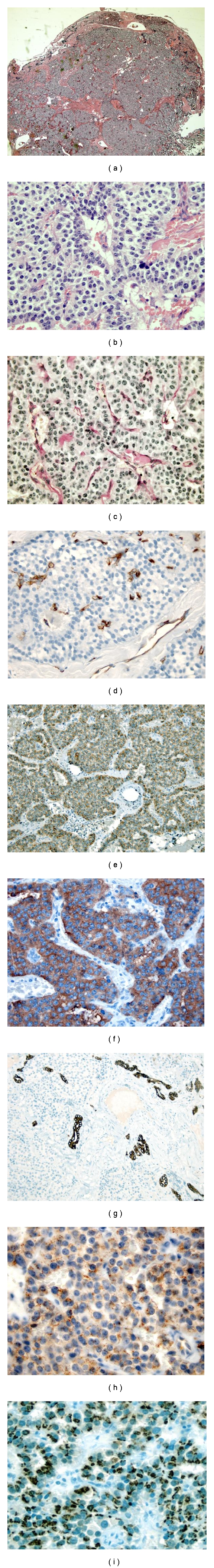
Histopathological analysis of the surgical specimen. (a) Hematoxylin and eosin staining (low magnification) showing gross appearance of the tissue and presence of a pseudocapsule with variable thickness. (b) Hematoxylin and eosin staining (200x) showing monomorphic cells with abundant granular cytoplasm and central nuclei, in contact with capillary basement membranes, stained with Period-Acid Schiff (PAS) reaction (c, 200x). (d) Capillaries are stained with anti-CD34 (200x). The strong chromogranin (e) and Synaptophysin (f) immunoreactivity indicates a neuroendocrine origin, while the few areas staining for cytokeratin-7 (g) are residual exocrine tissue (100x). A visual comparison between insulin (h) and proinsulin (i) staining (200x) suggests a stronger proinsulin immunoreactivity.

The sample was fixed in formalin and sections stained with hematoxylin and eosin and Schiff period acid reaction (to visualize capillary basement membranes) using standard commercially available kits. Immunohistochemistry was performed with antibodies directed against CD34 (to stain capillaries), chromogranin and synaptophyin (neuroendocrine tumor markers), insulin, and proinsulin (clone GS9A8, Developmental Studies Hybridoma Bank, University of Iowa). The nodule was composed of a solid tumor with a pseudocapsule, made up of confluent growing areas of highly vascularized endocrine pancreatic tissue. Neoplastic cells were monomorphic, with abundant granular cytoplasm and central nuclei; they were in close contact with capillary basement membranes (reminiscent of an endocrine nature). Strong staining for chromogranin and synaptophyin confirmed the neuroendocrine origin of the tissue. Insulin staining was weak and inhomogeneous among cells, while proinsulin staining was stronger and more homogenous (Figure 2).

### 2.5. Followup

Four weeks after the removal of the nodule, proinsulin (0.5 pmol/L; 4.0 pg/mL) and insulin (15 pmol/L (2.1 mU/L)) concentrations were normal. At the last telephone contact, 3 months after surgery, the patient had no more complained hypoglycemic symptoms.

## 3. Discussion

The clinical history of this patient was highly suggestive of an organic hypoglycemia. However, the low plasma insulin concentrations, in relation to glucose levels, induced caution in referring the patient to the surgeon and an extensive imaging workup was performed. In fact, even with dedicated imaging techniques, accessory spleens have been sometimes confused with insulinomas and vice versa [3, 4]. The high proinsulin concentrations could be indicative of the presence of a proinsulinoma; importantly, the plasma proinsulin assay used does not cross-react with intact human insulin or other proinsulin fragments. It has been previously pointed out that the use of highly specific plasma insulin immunoassays reduce the chances of identifying patients with high proinsulin concentrations and possible proinsulinomas [5, 6]. However, proinsulin-only secreting tumors are very rare (a few cases are described in the literature [6–9]) while pancreatic endocrine tumors usually produce and secrete both insulin and proinsulin [10, 11]. Anatomical diagnosis was finally confirmed with selective angiography, the most invasive and accurate approach, providing information of vascular density and origin. Remarkably, there was no increase in proinsulin concentrations after calcium stimulation, in agreement with the observation that proinsulin is much less responsive to this test than insulin [12]. This observation may underline that adenomas with proinsulin-only or predominant proinsulin over insulin secretion are less responsive to calcium stimulation, likely because of their more immature nature compared with typical insulinomas. A detailed pr-operative clinical and imaging characterization is critical crucial to guide surgery. Octreotide scintigraphy was not considered for its low negative predictive value in the diagnosis of insulinomas, while labeled exendin-4 [13] and ^18^F L-DOPA positron emission tomography were not routinely available, and their value has yet to be confirmed in the diagnosis of insulinomas [14, 15].

In this case, an enucleative conservative approach lead to a favorable prognosis, without exposing the patient to larger pancreatic resection and risk of developing diabetes.

Histopathological analysis confirmed the classic neuroendocrine nature of the adenoma, with blunted insulin immunoreactivity and higher proinsulin content. Remarkably, the GS-9A8 antibody clone used for proinsulin immunostaining does not cross-react with human insulin or c-peptide, providing an unbiased picture of proinsulin content. Finally, the resolving of symptoms and the drop in proinsulin concentrations after the removal of the tumor confirmed the diagnosis of proinsulinoma.

## Figures and Tables

**Table 1:** Daytime glucose, insulin and c-peptide variations during hospital stay. Insulin and c-peptide were measured using automated immunoassays from American Systems DPS (Diagnostic Products Corporation) optimized on a Siemens Immulite 2000^©^ platform.

	Glicemia mmol/L (mg/dL)	Insulina pmol/L (mU/L)	c-peptide pmol/L (*μ*g/L)
Before breakfast	1.9 (34)	39 (5.4)	0.60 (1.8)
After breakfast	5.2 (94)	79 (11.0)	1.12 (3.4)
Before lunch	3.0 (54)	62 (8.6)	0.82 (2.5)
After lunch	7.7 (139)	113 (15.7)	1.56 (4.7)
Before dinner	3.3 (59)	80 (11.1)	1.23 (3.7)
After dinner	4.9 (88)	54 (7.5)	0.99 (3.0)

**Table 2:** Results of the venous sampling during selective angiography (Imamura test). Normal range for insulin concentration was 43–210 pmol/L (6.0–29.1 mU/L) and for proinsulin concentration was <1.1 pmol/L (10 pg/mL).

		Time after Ca^2+^-gluconate bolus			
Artery	Analyte	0′	30′	60′	120′
Splenic	Insulin, pmol/L (mU/L)	55 (7.6)	55 (7.6)	62 (8.6)	67 (9.3)
	Proinsulin, pmol/L (pg/mL)	20 (185)	20 (176)	14 (131)	20 (176)
	Glucose, mmol/L (mg/dL)	3.0 (54)	3.1 (55)	2.8 (51)	2.9 (53)
Distal splenic	Insulin	43 (6.0)	32 (4.4)	46 (6.4)	65 (9.0)
	Proinsulin	18 (155)	16 (145)	15 (132)	27 (234)
	Glucose	3.0 (54)	2.4 (43)	2.7 (49)	3.3 (60)
Superior mesenteric	Insulin	32 (4.5)	<14 (2.0)	60 (8.3)	43 (6.0)
	Proinsulin	19 (166)	22 (192)	22 (196)	24 (212)
	Glucose	2.6 (47)	2.1 (37)	3.1 (55)	3.3 (60)
Gastroduodenal	Insulin	53 (7.4)	37 (5.1)	38 (5.3)	42 (5.8)
	Proinsulin	19 (166)	20 (179)	18 (163)	18 (155)
	Glucose	2.6 (47)	2.8 (50)	3.2 (58)	3.1 (56)
